# Study on the material basis and immunological enhancement activity of dangdi oral liquid

**DOI:** 10.1016/j.heliyon.2024.e32160

**Published:** 2024-05-29

**Authors:** Zhihong Zhou, Minzhuo Liu, Xin Zhao, Haixia Li, Qin Hu, Zhiping Jiang

**Affiliations:** a Department of Pharmacy, Hunan Children's Hospital, Changsha, 410007, China; b College of Biological and Chemical Engineering, Changsha University, Changsha, 410022, China; c Traditional Chinese Medicine department, Hunan Children ’s Hospital, Changsha, 410007, China

**Keywords:** Dangdi oral liquid (DDO), Bioactive substances, UPLC-Q-TOF/MS, Immunity

## Abstract

Studies have shown that a lot of traditional Chinese medicines could improve the immunity of the body. Dangdi oral liquid (DDO) was mainly composed of *Angelica sinensis* (Oliv.) Diels (Danggui), *Rehmannia glutinosa* Libosch. (Dihuang), *Achyranthes bidentata* Bl. (Niuxi), *Glycyrrhiza uralensis* Fisch. (Gancao). In this study, the rapid ultra-performance liquid chromatography coupled with quadrupole time-of-flight mass spectrometry (UPLC-Q-TOF/MS) method was used to identify the potentially effective compounds of DDO. Then the immune activity of DDO was measured by lymphocyte proliferation, macrophage phagocytic function, NK cell activity, delayed type hypersensitivity reaction, hemolytic plaque number, sIgA content and immune organ index. The results showed that a total of 51 compounds were identified. In addition, DDO could significantly promote the lymphocyte proliferation, improve macrophage phagocytic ability, NK cell activity, hemolytic plaque number, sIgA content and immune organ index compared with control group, and the medium dose possessed the best efficacy (P＜0.05). These results indicated that DDO could enhance the immunity of mice.

## 1 Introduction

Immunity is the common terminology in modern medicine. It is the physiological function of the body's immune system to recognize “oneself” and “non-self” and eliminate “non-self” in order to maintain the stability of the body's internal environment. Through immunity, the body can resist and eliminate the invasion of foreign pathogens, neutralize toxins, and eliminate the damage, senescence, and mutated cells in the body [1]. Immunity is the kind of active defense mechanism of human body, which is of great significance in maintaining physiological and vital activities [2]

Under the normal physiological conditions, the immune system of the body relies on its innate immunity and acquired adaptive immunity to play the immune function together, so that the physiological function of the body is kept in the relatively stable state. If the body's immune function is abnormal, it will lead to adverse consequences such as allergic reaction, low immunity and so on. Studies have proved that people with low immunity are more vulnerable to the diseases and are not easy to recover, in the long term, which will increase the risk of serious diseases in the population in the long run [3,4]

With the acceleration of the pace of modern life and the increasingly fierce social competition, the decline of immunity has gradually become the hidden danger in the life of modern people [5]. In addition, with the popularization of organ transplantation, the increase of the incidence of malignant tumors, and the appearance of the aging population, the number of people in the state of immune hypofunction keeps rising. Immunosuppression is particularly harmful to children and the elderly [6,7]. Therefore, the research and development of safe and efficient immunomodulatory drugs to improve people's immunity has important clinical significance

At present, most of the immune enhancers used in clinic are synthetic drugs, which not only improve the immunity of the body, but also have certain toxic and side effects. A number of studies have found that some traditional Chinese medicines have natural immune enhancement effects [8]. Traditional Chinese medicines (TCM) are mostly natural plants, which have some advantages of low drug resistance, little toxic and side effects, few adverse reactions and no dependence. In the process of disease prevention and treatment, predecessors accumulated a lot of experience in TCM compatibility, which laid the foundation for the research and development of TCM as immune enhancer. In recent years, with the development of immunopharmacology and chemistry of TCM and people's pursuit of quality of life, the research and development of new multi-effect immune enhancers is increasingly urgent. A large number of research results showed that TCM and ingredients could enhance and restore the immune function of the body, and had broad application prospects in the aspects of inflammation, anti-immune damage, anti-tumor, anti-aging and so on [[9], [10], [11], [12]].

Dangdi oral liquid (DDO) is a representative prescription based on the improvement of immunity in TCM theory, which has been clinically used for many years in the Hunan Children's Hospital. DDO was mainly composed of *Angelica sinensis* (Oliv.) Diels (Danggui), *Rehmannia glutinosa* Libosch. (Dihuang), *Achyranthes bidentata* Bl. (Niuxi), *Glycyrrhiza uralensis* Fisch. (Gancao). *Angelica sinensis* (Oliv.) Diels could nourish blood, promote blood circulation to relieve pain. *Rehmannia glutinosa* Libosch. could nourish Yin and supplement blood, supplement essence and marrow. *Achyranthes bidentata* Bl. Could tonify the liver and kidney, strengthen the muscles and bones. *Glycyrrhiza uralensis* Fisch. could tonify spleen and qi, expel phlegm and relieve cough, relieve heat and detoxification, alleviate medicinal properties. DDO has shown positive effects in clinical study, but its effective components and mechanism remain elusive. In the present study, the chemical compositions of DDO were analyzed by ultra-performance liquid chromatography coupled with quadrupole time-of-flight mass spectrometry (UPLC-Q-TOF/MS). Additionally, the immune activity of DDO was measured in 6 aspects, which were the measurement of cellular immune function (including lymphocyte proliferation test and delayed allergic reaction test); the measurement of humoral immune function (including antibody producing cell test); the measurement of mucosal immune function (sIgA content); the measurement of macrophage phagocytic function (including neutral red phagocytosis test and mouse carbon clearance test); the measurement of natural killer cell (NK cell) activity (lactate dehydrogenase LDH assay) and the measurement of immune organ index. This study not only clarifies the material basis of DDO to improve the immunity, but also provides the new ideas and theoretical foundation for future researching the pharmacological mechanism of DDO.

## 2 Materials and methods

### 2.1 Chemicals and reagents

Methanoic acid and acetonitrile (HPLC grade) were bought from Merck (Darmstadt, Germany). Methanol (HPLC grade) was bought from Fisher Scientific (Fisher, USA). Water for UPLC analysis was purified by the Milli-Q water purification system (Millipore, USA). Fetal bovine serum was bought from Thermo Fisher Scientific Inc. RPMI-1640 and DMEM medium was bought from Life technologies corporation (USA). Benzylpenicillin, streptomycin, Concanavalin A (ConA) and Lipopolysaccharide (LPS) were bought from Solarbio technology Co., Ltd. Dimethyl sulfoxide (DMSO) was produced by Sinopharm Chemical Reagent Co., Ltd. 3-(4,5-Dimethylthiazol-2-yl)-2, 5-diphenyltetrazolium bromide (MTT, Amresco Co.) was diluted into 5 mg/mL with phosphate-buffered saline (PBS, pH 7.4). These reagents were filtered through a 0.22 μm millipore filter. ConA and LPS solution was stored at −20 °C, MTT solution at 4 °C in dark bottle.

### 2.2 Animals and administration

Kunming mice (18–22 g) and SPF grade guinea pigs (300–350 g) were purchaed from Beijing HFK Bioscience Co., Ltd. (SCXK2022-0004, Beijing, China). The experimental protocol was approved by the Ethics Review Committee of Animal Care and Use of Hunan Children's Hospital (HCHDWLL-2021-10). All animals were feed in the room (22–25 °C with 50 % ± 10 % humidity) for one week, which had free access to water and standard laboratory food.

### 2.3 Preparation of DDO

DDO was mainly composed of Angelica sinensis (Oliv.) Diels (Danggui), Rehmannia glutinosa Libosch. (Dihuang), Achyranthes bidentata Bl. (Niuxi), Glycyrrhiza uralensis Fisch. (Gancao), which (No. 20220123) was prepared by China Resources Sanjiu Medical & Pharmaceutical CO., LTD, Shenzhen, China.

### 2.4 The characterization of chemical composition of DDO by UPLC-Q-TOF/MS

#### 2.4.1 Sample preparation

The sample was dissolved into a solution of 5 mg/mL with 70 % methanol, which was centrifugated at 20 000 r/min for 10 min. The supernatant was collected and filtered via 0.22 μm millipore membranes.

#### 2.4.2 Chromatographic conditions

Chromatographic analysis was performed on an ACQUITY UPLC system (Waters, Milford, USA). Chromatographic separation was carried out on an ACQUITY UPLC HSS C_18_ column (2.1 mm × 100 mm, 1.7 μm), the temperature of column was 40 °C, the flow rate was 0.3 mL/min, and the injection volume was 2 μL. The mobile phase was composed with 0.1 % formic acid-water solution-5 mM ammonium formate (A) and acetonitrile (B) in a gradient elution. The program was as follows: 0–1 min, 2 % B; 1–22 min, 2%–95 % B; 22–26.4 min, 95 % B; 26.4–26.5 min, 95%–2% B; 26.5–30 min, 2 % B.

#### 2.4.3 MS spectrometry conditions

The MS analysis was proceed by Xevo G2-XS Q-TOF/MS (Waters Corp.,USA) with an electrospray ionisation (ESI) source. The optimal conditions were as follows: capillary voltage at 3.0 kV and 2.5 kV in positive and negative ion mode respectively; source temperature at 100 °C; desolvation temperature at 250 °C; sampling cone voltage at 40 V; the flow rate of cone gas and desolvation gas (N_2_) were set at 50 L/h and 600 L/h respectively. MSE scanning mode detection was used, leucine-enkephalin was used as correction solution, and the collected data was calibrated by locking mass function. The collision energy of low energy channel was 6 V, and the collision energy of high energy channel was 30–65 V. The mass scan range was set from 100 to 1000 *m*/*z*.

#### 2.4.4 Establishment of chemical composition database and data processing

The system database, analysis platform of TCM (TCMSP, https://old.tcmsp-e.com/tcmsp.php), PubChem (https://pubchem. Ncbi. nlm.nih.gov) and the literature about the chemical composition information including name, molecular formula, the structural formula in each compounds of DDO were collected, and then the database of chemical composition was established, it was imported into UNIFI 2.0 software. The mass spectrum data collected from positive and negative ion modes were automatically matched with the database in UNIFI software, and the compounds with the deviation of 2 mDa were selected, then they were identified and verified, and the final results were obtained.

### 2.5 The effect of DDO on splenic lymphocyte proliferation in vitro

The spleen from Kunming mice was collected under aseptic conditions, and placed in the small dish containing the sterile PBS. The spleen was gently mashed to make the cell suspension. They were filtered through a 200-mesh screen, then washed twice with PBS, centrifuged at 1000 r/min for 10 min. Then the cells were suspended with RPMI-1640 contained fetal bovine serum, and the number of viable cells was counted by trypan blue staining (which should be above 95 %), and the cell concentration was adjusted to 1 × 10^6^ cells/mL. The lymphocytes suspension was divided into two parts: one was added with ConA or LPS, and respectively incubated into 96-well plates, 100 μL/well. Then, DDO were added at different concentration from 125 to 7.81 μg/mL. In cell control group and ConA/LPS control group, RPMI-1640 medium and ConA/LPS were added respectively, the total volume was 200 μL/well. Repeat wells were set for each concentration. The final concentration of ConA or LPS reached to 5 μg/mL. After 44 h in an incubator at 5 % CO_2_ and 37 °C, 20 μL of MTT (5 mg/mL) was added to each well, and cultivated for 4 h. Then the supernatant was removed and 100 μL of DMSO was added to each well. The plate was shaken to dissolve the crystal completely [13]. The absorbance value was measured at the wave length of 570 nm by microplate reader (Model 680, Bio-Rad, USA).

### 2.6 Neutral red phagocytosis experiment

The macrophages RAW264.7 were added to 96-well cell plates, the number was 1 × 10^5^ cells per well. After monolayers were grown, the culture medium was discarded and washed with PBS for twice. Then, each concentration of drug diluted with culture medium (125, 62.5, 31.25, 15.63, 7.81 μg/mL) were added, 100 μL/well, with 4 replicates. Control group (100 μL medium) were designed. After cultivated for 24 h, the culture medium was discarded and 1 mg/mL neutral red normal saline solution was added for another 2 h. Then the supernatant was discarded and washed. 100 μL of cell lysis solution (ethanol: acetic acid = 1:1) was added to each well, and placed for 2 h at room temperature. After the cells were dissolved, the absorbance was measured at 540 nm [14]. Phagocytosis rate is calculated according to the following formula: phagocytosis rate = the absorbance value of experimental group/the absorbance value of control group.

### 2.7 Detection of antibody-producing cells—hemolytic plaque assay [15]

#### 2.7.1 The preparation of sheep red-blood cell (SRBC)

Sheep's jugular vein blood was collected aseptically. The blood was put into a sterilized conical flask with glass beads and shaken in one direction for 20 min. The defibrillated blood was placed in a 15 mL centrifuge tube and stored in a refrigerator at 4 °C for future use.

#### 2.7.2 Preparation of complement

The blood of guinea pigs was collected through abdominal aorta, and the serum was separated. Then 1 mL of SRBC was added into 5 mL of guinea pig serum, placed in refrigerator at 4 °C for 30 min, then the serum was centrifuged, the supernatant was collected and stored at −70 °C. When used, it was diluted by SA bufferat 1:8.

#### 2.7.3 Animals and drug administration

Forty female Kunming mice (18–22 g) were randomly divided into 4 groups with 10 mice in each group. Five times (4 g/kg), 10 times (8 g/kg) and 30 times (24 g/kg) of the recommended dose of human body were used as low-dose, medium-dose and high-dose groups, and negative control group was also set. The administration group was given 0.4 mL of DDO with different concentration intragastrically every day, negative control group was given 0.4 mL of normal saline, once a day, for 30 days. 24 h after the last administration, each mouse was injected intraperitoneally with 0.2 mL of 2 % SRBC suspension.

#### 2.7.4 Preparation of splenic cell suspension

Mice immunized with SRBC for 4 days were sacrificed for cervical vertebrae dislocation, placed in 75 % alcohol, separated the spleen aseptically, then placed in a small dish containing PBS, gently ground the spleen to make cell suspension, filtered through a 200-mesh screen, centrifuged at 1000 r/min for 10 min, washed twice with PBS, and finally the cells were suspended in 5 mL PBS. The cell concentration was adjusted to 5 × 10^6^ cells/mL.

#### 2.7.5 Determination of plaque

Agarose solution (0.5 g agarose was dissolved with distilled water to 100 mL) was added to the 6-well plate, 1 mL/well, and it was quickly shaked in the same direction to smooth it. 1 g of agarose was dissolved with distilled water to 100 mL, then the medium was kept in the 45–50 °C water bath, mixed with equal amount of Hank's solution, then divided into small test tubes, 0.5 mL per tube, and then added 50 μL of 10 % SRBC (v/v, prepared with SA buffer solution) and 20 μL of spleen cell suspension (5 × 10^6^ cells/mL) into the tube. They were quickly mixed and poured on 6-well plate with agarose thin layer. After agarose solidification, the 6-well plate was incubated in carbon dioxide incubator for 1.5 h, and then the complement diluted with SA buffer (1:8) was added to the 6-well plate. After incubation for 1.5 h, the number of hemolystic plaques was counted.

### 2.8 Determination of NK cell activity in mice ([16])

#### 2.8.1 YAC-1 cells (target cells)

YAC-1 cells were subcultured 24 h before the experiment. Before use, the cells were washed 3 times with PBS, and the cell concentration was adjusted to 4 × 10^5^ cells/mL with RPMI1640 culture solution.

#### 2.8.2 Animals and drug administration

The administration of mice was consistent with 2.7.3 part.

#### 2.8.3 Preparation of splenic cell suspension (effector cells)

24 h after the last administration, the mice were sacrificed and placed in 75 % alcohol. The spleen was extracted aseptically and placed in a small dish containing an appropriate amount of sterile PBS. The spleen was gently ground to make the cell suspension. It was filtered through a 200-mesh screen, washed twice with PBS, centrifuged 1000 r/min for 10 min. The supernatant was discarded and 2 mL of red cell lysis buffer (NH_4_Cl-Tris) was added to lyse the red cells. After re-suspension, it was stood for 2min. RPMI1640 containing 10 % fetal bovine serum was added and centrifuged at 1000 r/min for 10 min. The supernatant was discarded and the sediment was resuspended with 1 mL RPMI1640, and the number of viable cells was counted by Trypan blue staining (which should be above 95 %), and the cell concentration was adjusted to 2 × 10^7^ cells/mL, the splenic cells were prepared.

#### 2.8.4 Detection of NK cell activity

100 μL of target cells and effector cells (effector:target was 50:1) were added into 96-well culture plates. Target cells naturally released well added 100 μL target cells and culture medium, and the maximum release well of target cell added 100 μL of target cells and 1 % NP40 respectively. Three parallel wells were set for each well, and the cells were cultured in a 5 % CO_2_ incubator at 37 °C for 4 h. Then, the cell culture medium in the 96-well culture plate was sucked out and placed into the 1.5 mL centrifuge tube for 5 min at 1500 r/min. 100 μL of superneant was sucked from each tube and placed into the new 96-well culture plate, and 100 μL of LDH solution was added. After reaction for 3 min, 30 μL of HCl (1 mol/L) was added to each well, and the absorbance was measured at 490 nm. According to the following formula, when the NK cell activity of the tested sample group was significantly higher than that in the control group, the result of this experiment could be determined as positive.

The NK cell activity (%) = (the absorbance value of experimental group - the absorbance value of natural release control group)/(the absorbance value of maximum release control group - the absorbance value of natural release control group) × 100 %.

### 2.9 Delayed-type hypersensitivity (DTH) reaction test ([17])

#### 2.9.1 Preparation of 1 % dinitrofluorobenzene (DNFB) solution for sensitization

Weighing DNFB 200 mg, and put it into a clean vial, 20 mL of acetone and sesame oil solution (acetone: sesame oil was 1:1) was poured in the vial, and mixed well for using

#### 2.9.2 Animals and drug administration

The administration of mice was consistent with 2.7.3 part. 24 h after the last administration, hair was removed from the abdomen of each mouse, the range was about 3 cm × 3 cm, and the skin should not be damaged. Surgical tape was used to carefully clear the remaining hair fragments, and 50 μL 1 % DNFB solution was applied according to the above grouping method.

#### 2.9.3 Determination of DTH reaction

On day 5 after sensitization, 20 μL of 1 % DNFB solution was evenly applied to both sides of the right ear. 24 h after administration, the mice were sacrificed, weighed, the left and right auriculae were cut, and the ear pieces removed with the 6 mm diameter perforator were weighed. The difference between the weights of the left and right ear pieces was the degree of swelling. The swelling rate = (the right ear weight - the left ear weight)/left ear weight × 100 %.

### 2.10 Carbon clearance test in mice

The administration of mice was consistent with 2.7.3 part. 24 h after the last administration, dilute Indian ink (diluted 8 times with normal saline) was injected from the tail vein of mice according to the weight (0.1 mL/10g). At the 2 min and 10 min after injection of ink, 20 μL of blood was collected from the angular vein and immediately added to 2 mL of 0.1%Na_2_CO_3_ solution. The optical density (OD) was measured at 600 nm wavelength by spectrophotometer, and Na_2_CO_3_ solution was used as blank control [18]. The mice were sacrificed, the liver, thymus and spleen were taken, the blood stains on the surface of the organs were dried with filter paper, and then they were weighed separately. The immune organic index was calculate according the formula: organic index = the weight of immune organ (mg)/the weight of body (g) [19]

Phagocytosis index was used to indicate the carbon clearance ability of mice. The formula to calculate phagocytosis index as follows

K=(lgOD_1_-lgOD_2_)/(t_2_-t_1_); Phagocytosis index = {(body weight)/(liver weight + spleen weight)} × K^1/3^. OD_1_ was OD at 2 min; OD_2_ was OD at 10 min; t_1_, 2 min; t_2_, 10 min.

### 2.11 The effect of DDO on splenic lymphocyte proliferation in vivo

The administration of mice was consistent with 2.7.3 part. 24 h after the last administration, the mice were sacrificed and placed in 75 % alcohol. The spleen was extracted aseptically and placed in a small dish containing an appropriate amount of sterile PBS. The spleen was gently ground to make the cell suspension. It was filtered through a 200-mesh screen, washed twice with PBS, centrifuged 1000 r/min for 10 min. Then the cells were suspended in complete culture medium, and the number of viable cells was counted by Trypan blue staining (which should be above 95 %), and the cell concentration was adjusted to 1 × 10^6^ cells/mL, the splenic lymphocytes were prepared. The preparation of splenic lymphocytes was same to 2.5 part. Splenic lymphocytes were mixed with equal volume RPMI-1640 medium, LPS and ConA respectively, and then inoculated into 96-well plates, the total volume was 200 μL/well, with 4 repeat for each sample. After 44 h culture in an incubator at 37 °C and 5 % CO_2_, 20 μL of MTT was added to each well, and continued to culture for 4 h. Then 100 μL of dimethyl sulfoxide was added to each well to dissolve the crystal. The absorbance value of each well was measured at 570 nm by microplate reader.

### 2.12 Effect of DDO on sIgA level in immunosuppression mice

#### 2.12.1 Animal and drug administration

Fifty female Kunming mice (18–22 g) were randomly divided into 5 groups with 10 mice in each group. Five times (4 g/kg), 10 times (8 g/kg) and 30 times (24 g/kg) of the recommended dose of human body were used as low-dose, medium-dose and high-dose groups, modeling group (cyclophosphamide) and negative control group was also set. The administration group was given 0.4 mL of DDO with different concentration intragastrically every day, negative control group was given 0.4 mL of normal saline, once a day, for 28 days. On days 24, 25 and 26, mice in the modeling group and the administration group were intraperitoneally injected 100 mg/kg cyclophosphamide, once a day for 3 consecutive days to create immunosuppression model. The negative control group was intraperitoneally injected sterile PBS [20].

#### 2.12.2 The extract of sIgA in respiratory tract and digestive tract

On the 28th day, 5 mice in each group were randomly selected to expose the trachea, the syringe needle was inserted to the trachea parallel, then clamped the needle with tweezers, and rinsed with precooled PBS, the rinsing liquid was collected and centrifuged at 1500 rpm/min for 10 min at 4 °C. The supernatant was collected and stored at −80 °C. Opening the abdominal cavity of mouse, the ileum was collected, and the contents were removed gently. After weighing the ileum, the precooled PBS was added to make the tissue concentration of the sample 50 mg/mL. The sample was homogenized immediately on ice, and then centrifuged at 4000 rpm/min for 15 min at 4 °C. The supernatant was collected and stored at −80 °C.

#### 2.12.3 Determination of sIgA

The level of sIgA in respiratory tract and digestive tract was measured by ELISA kit according the instruction.

### 2.13 Statistical analysis

The results were presented as mean ± SD (standard deviation). The statistical significance was analyzed by student's test using SPSS 23.0 for mean differences among the samples. Differences between groups at p < 0.05 were considered statistically significant.

## 3 Results

### 3.1 Main chemical composition of DDO

**Fig. 1:**
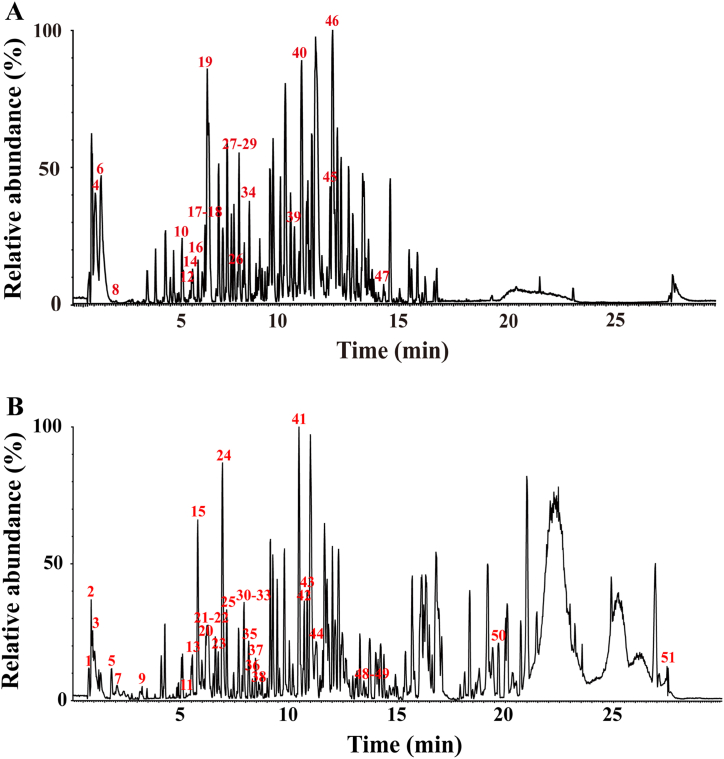
Basic peak ion flow (BPI) chromatograms. (A) BPI in negative ion models. (B) BPI in positive ion models. Peak 1–51 represents the different compounds which were listed in Table 1.

**Fig. 2:**
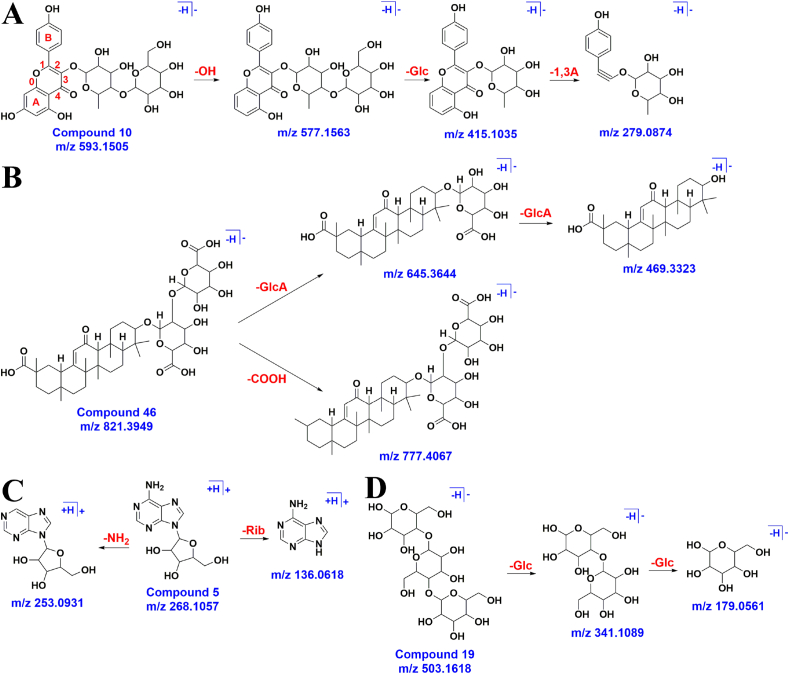
Possible fragmentation pathways of rosaceine b, glycyrrhizic acid, adenosine and β-amylase. (A) Rosaceine b. (B) Glycyrrhizic acid, (C) Adenosine. (D) β-amylase.

The ion flow chromatograms obtained based on the established method to study the composition of DDO were shown in (Fig. 1, Fig. 2) for positive and negative ion patterns, respectively. From the graph, it could be seen that the peak shape in the detection conditions was more ideal, and the distribution was relatively uniform. It shows that the mass spectrometry system had good stability in this experiment, and the obtained experimental data were stable and reliable, which could provide data support and a theoretical basis for the subsequent experimental results.

### 3.2 Identification of the compounds in DDO

Under the optimized chromatographic and MS parameters, 51 compounds in DDO were identified (Table 1). 51 compounds included 26 flavonoids, 5 triterpenoids, 5 alkaloids, 4 glycosides, 3 coumarins, 2 organic acids, 2 esters and 4 other compounds. The specific information of compounds is shown in Table 1.

**Table 1:** Identification of the compounds in DDO by UPLC-Q-TOF/MS in both negative and positive ion models.

Peak	t_R_ (min)	Formula	Mode	Theory *m*/*z*)	Measured *m*/*z*	Error	Fragmentation ion attribution	Adducts	Identification	Type
1	0.87	C_6_H_14_N_4_O_2_	+	174.1117	175.1183	−0.6	158.0924[M − NH_2_]^+^	+H	Arginine	A
2	0.91	C_12_H_22_O_11_	+	342.1162	365.1055	0.1	204.0992[M-C_3_H_6_O_6_]^+^	+Na	Maltose	E
3	1.06	C_12_H_22_O_11_	+	342.1162	365.1055	0	265.0918[M-C_2_H_5_O_3_]^+^	+Na	Sucrose	E
4	1.32	C_6_H_8_O_7_	–	192.0270	191.0199	0.2	173.0092[M − OH]^-^	-H	citric acid	G
5	1.82	C_10_H_13_N_5_O_4_	+	267.0968	268.1057	1.6	253.0931[M − NH_2_]^+^; 136.0618[M-Rib]^+^	+H	Adeninenucleoside	D
6	1.85	C_12_H_22_O_11_	–	342.1162	387.1137	−0.7	281.0878 [M-2CH_2_OH]^-^	+HCOO, –H	α-kojibiose	E
7	1.99	C_5_H_5_N_5_O	+	151.0494	152.0568	0.1	135.0301 [M − NH_2_]^+^	+H	Guanine	D
8	2.01	C_10_H_13_N_5_O_5_	–	283.0917	282.0843	−0.1	179.0574 [M-C_3_H_6_NO_3_]^-^	-H	Guanosine	D
9	3.28	C_21_H_24_O_8_	+	404.1471	405.1562	1.8	195.0288[M-C_12_H_17_O_3_]^+^	+H	Citromitin	B
10	5.1	C_27_H_30_O_15_	–	594.1585	593.1505	−0.7	415.1035[M-glc-OH]^-^; 577.1563[M − OH]^-^;	-H	multiflorin b	B
							279.0874[M-Glc-1.3A]^-^			
11	5.34	C_26_H_28_O_14_	+	564.1479	565.1565	1.4	231.0652[M-C_13_H_17_O_10_]^+^; 257.0808 [M-C_11_H_15_O_10_]^+^	+H	Schaftoside	B
12	5.41	C_33_H_42_O_19_	–	742.2320	787.2320	1.8	433.1140 [M-2Glc]^-^; 271.0612[M-3Glc]^-^	+HCOO	Naringenin-4′-glucoside-7-neohesperidoside	B
13	5.42	C_27_H_32_O_14_	+	580.1792	581.1859	−0.5	273.0758[M-Glc-Rha]^+^	+H	Naringin	B
14	5.73	C_27_H_32_O_14_	–	580.1792	579.1739	1.9	255.0663[M-2Oglc]^-^	-H	Narirutin	B
15	5.8	C_22_H_28_O_11_	+	468.1632	491.1510	−1.4	307.1176 [M-Glc]^+^; 259.0601[M-CH2OGlc-CH_3_]^+^; 235.0601[M-Glc–CH_3_–C_3_H_3_O]^+^	+Na	prim-o-beta-*d*-glucosylcimifugin	B
16	5.81	C_9_H_8_O_3_	–	164.0473	163.0409	0.9	150.0322 [M − CH_2_]	-H	4-Acetylbenzoic acid	G
17	6.14	C_20_H_20_O_8_	–	388.1158	433.1146	0.5	283.0612[M-C_4_H_9_O_3_]^-^; 311.0561[M-C_3_H_9_O_2_]^-^	+HCOO	3,4,6-trihydroxyphenanthrene-3-o-β-*d*-gluco-pyranoside	A
18	6.15	C_22_H_22_O_10_	–	446.1213	491.1206	1.1	283.0612[M-Oglc]^-^; 268.0377[M-Oglc-CH_3_]^-^;	+HCOO, –H	Rhamnocitrin 3-rhamnoside	B
							285.0405[M-Glc-OH]^-^			
19	6.21	C_18_H_32_O_16_	–	504.1690	503.1618	0.4	341.1089[M-Glc]^-^; 179.0561[M-2Glc]^-^	-H	Amylase	E
20	6.22	C_26_H_30_O_13_	+	550.1686	573.1572	−0.7	419.1337[M-C_5_H_7_O_4_]^+^; 451.1024[M-C_2_H_11_O_4_]^+^	+Na, +H	liquiritigenin-7-*O*-d-apiosyl-4′-*O*-D-glucoside	B
21	6.28	C_21_H_22_O_9_	+	418.1264	441.1176	2	257.0808[M-Glc]^+^; 137.0233[M-C_14_H_17_O_6_]^+^;	+Na, +H	Isoliquiritin	B
							239.0703[M-Oglc]^+^			
22	6.28	C_18_H_14_O_6_	+	326.0790	349.0701	1.8	137.0233[M-C_11_H_9_O_3_]^+^; 295.0601[M-OCH_3_]^+^;	+Na	ophiopogonone a	B
							177.0546[M–OH–C_8_H_4_O_2_]^+^			
23	6.75	C_15_H_12_O_5_	+	272.0685	273.0758	0.1	153.0182[M-C_8_H_7_O]^+^; 163.0390[M-C_6_H_5_O_2_]^+^;	+H	(2R)-5,7-dihydroxy-2-(4-hydroxyphenyl)chroman-4-one	B
							147.0441[M-C_6_H_5_O_3_]^+^			
24	6.93	C_22_H_28_O_10_	+	452.1682	475.1555	−1.9	243.0652[M-Oglc-2CH_3_]^+^; 216.0417[M-C_10_H_20_O_6_]^+^; 291.1227[M-Glc]^+^	+Na	(2S)-4-methoxy-7-methyl-2-[1-methyl-1-[(2S,3R,4S,5S,6R)-3,4,5-trihydroxy-6-methylol-tetrahydropyran-2-yl]oxy-ethyl]-2,3-dihydrofuro[3,2-g]chromen-5-one	B
25	7.14	C_28_H_34_O_15_	+	610.1898	611.1957	−1.4	303.0863[M-Rha-Glc]^+^	+H, +Na	(2S)-7-[(2S,3R,4S,5S,6R)-4,5-dihydroxy-6-methylol-3-[(2S,3R,4R,5R,6S)-3,4,5-trihydroxy-6-methyl-tetrahydropyran-2-yl]oxy-tetrahydropyran-2-yl]oxy-5-hydroxy-2-(3-hydroxy-5-methoxy-phenyl)chroman-4-one	B
26	7.55	C_28_H_32_O_14_	–	592.1792	591.1737	1.8	293.0667[M-Oglc–OH–C_8_H_10_O]^-^; 525.1402[M-3OH–CH_3_]^-^; 469.1352[M–OH–C_7_H_6_O]^-^	-H	Linarin	B
27	7.65	C_15_H_10_O_4_	–	254.0579	253.0499	−0.7	161.0244[M-C_6_H_5_O]^-^; 177.0193[M − C_6_H_5_]^-^;	-H	Primetin	B
							237.0557[M − OH]^-^			
28	7.66	C_21_H_22_O_9_	–	418.1264	417.1183	−0.8	374.0643[M-3CH_3_]^-^; 399.1085[M − OH]^-^;	-H	atsudaidai	B
							193.0506[M-C_11_H_13_O_5_]^-^			
29	7.66	C_21_H_22_O_9_	–	418.1264	417.1183	−0.8	255.0663[M-Glc]^-^	-H	Liquiritin	B
30	7.93	C_20_H_24_O_9_	+	408.1420	431.1310	−0.3	320.0891[M–CH_2_OH–C_3_H_6_O]^+^; 177.0546[M-Glc-C_4_H_3_]^+^	+Na	Ammijin	F
31	7.93	C_16_H_12_O_4_	+	268.0736	269.0797	−1.2	253.0495[M − CH_3_]^+^; 237.0546[M-CH_2_OH]^+^;	+H	Tectochrysin	B
							255.0652[M − CH]			
32	7.93	C_22_H_22_O_9_	+	430.1264	453.1137	−2	269.0808[M-Glc]^+^; 253.0495[M-Glc-CH_3_]^+^;	+Na	Ononin	B
							237.0546[M-Glc-OCH_3_]^+^			
33	7.96	C_18_H_19_NO_4_	+	313.1314	336.1216	1	177.0546[M-C_8_H_10_O]^+^	+Na	n-trans-feruloyltyramine	D
34	8.13	C_9_H_10_N_2_O_4_	–	210.0641	255.0622	0	193.0381[M − NH_3_]^-^	+HCOO	PHP- B	D
35	8.16	C_15_H_12_O_4_	+	256.0736	257.0823	1.5	137.0233[M-C_8_H_7_O]^+^; 147.0441[M-C_6_H_5_O_2_]^+^;	+H	Isoliquiritigenin	B
							163.0390[M-C_6_H_5_O]^+^			
36	8.32	C_16_H_18_O_5_	+	290.1154	291.1215	−1.2	243.0652[M-2CH_3_–OH]^+^; 205.0495[M-C_5_H_9_O]^+^; 217.0495[M-C_4_H_9_O]^+^	+H, +Na	5-*o*-methylvisamminol	B
37	8.47	C_16_H_12_O_5_	+	284.0685	285.0765	0.7	269.0445[M − CH_3_]^+^; 253.0495[M-OCH_3_]^+^;	+H, +Na	Acacetin	B
							161.0597[M-C_6_H_3_O_3_]^+^			
38	8.61	C_15_H_16_O_5_	+	276.0998	277.1080	0.9	205.0495[M-C_4_H_7_O]^+^; 259.0965[M − OH]^+^;	+H	Hamaudol	B
							229.0495[M-2CH_3_–OH]^+^			
39	10.2	C_15_H_12_O_4_	–	256.0736	255.0646	−1.6	165.0557[M-C_6_H_3_O]^-^	-H	Liquiritigenin	B
40	10.59	C_16_H_12_O_4_	–	268.0736	267.0655	−0.7	252.0428[M − CH_3_]^-^; 251.0350[M − CH_3_]^-^	-H	Formononetin	B
41	10.66	C_30_H_46_O_4_	+	470.3396	471.3458	−1.1	439.3571[M-2OH]^+^; 407.3308[M–COOH–OH]^+^	+H	18beta-glycyrrhetinic acid	C
42	10.71	C_21_H_22_O_8_	+	402.1315	403.1398	1.1	373.0918[M-2CH_3_]^+^; 343.0448[M-4CH_3_]^+^	+H, +Na	NO-biletin	B
43	10.74	C_38_H_56_O_11_	+	688.3823	689.3900	0.5	627.3164[M-C_3_H_9_O]^+^; 189.1638[M-C_24_H_35_O_11_]^+^; 217.1587[M-C_23_H_35_O_10_]^+^	+H	Methylcimicifugoside	C
44	11.35	C_21_H_20_O_6_	+	368.1260	369.1329	−0.4	200.0468[M-C_9_H_12_O_3_]^+^; 351.1227[M − OH]^+^;	+H	Suchilactone	H
							213.0546[M-C_8_H_11_O_3_]^+^			
45	11.85	C_39_H_62_O_14_	–	754.4140	799.4104	−1.8	631.3852[M-C_3_H_7_O_5_]^-^; 484.2314[M-C_15_H_26_O_4_]^-^; 495.2236[M-C_14_H_27_O_4_]^-^	+HCOO	ophiogenin-3-o-α-*l*-rhamnopyranosyl(1 → 2)-β-*d*-glucopyranoside	C
46	11.96	C_42_H_62_O_16_	–	822.4038	821.3949	−1.6	646.3717[M-GlcA]^-^; 470.3396[M-2GlcA]^-^;	-H	Glycyrrhizin	C
							778.4140[M-COOH]^-^			
47	13.32	C_20_H_20_O_5_	–	340.1311	339.1257	1.9	285.1132[M-C_3_H_3_O]^-^; 216.0428[M-C_8_H_12_O]^-^;	-H	Denudatin A	A
							255.0299[M − C_6_H_13_]^-^			
48	13.44	C_19_H_20_O_6_	+	344.1260	367.1171	1.9	281.0445[M-C_3_H_11_O]^+^; 241.0495[M-C_5_H_11_O_2_]^+^; 255.0652[M-C_4_H_9_O_2_]^+^	+Na	(3's)-hydroxydeltoin	F
49	13.45	C_45_H_72_O_16_	+	868.4820	891.4696	−1.7	181.0707[M-2Rha-C_27_H_47_O_2_]^+^; 557.3320[M-Rha-C_10_H_11_O_2_]^+^	+Na, +H	Dioscin	C
50	19.62	C_35_H_36_N_4_O_5_	+	592.2686	593.2762	0.3	547.2340[M–OH–C_2_H_5_]^+^; 533.2547[M-CH_2_COOH]^+^; 517.2234[M–OH–OCH_3_-C_2_H_5_]^+^	+H	PHA	H
51	27.53	C_12_H_22_O	+	182.1671	183.1743	0	154.1352[M − C_2_H_5_]^+^; 167.1430[M − CH_3_]^+^	+H	2-butyl-2-Octenal	A

Notes: A is other compound, B is flavonoid, C is triterpenoid, D is alkaloids, E is glycoside, F is coumarin, G is organic acid, H is ester.

### 3.3 Identification and fragmentation regularity of main compounds

#### 3.3.1 Flavonoids

A total of 26 flavonoids were characterized. Taking compound 10 as an example, in the negative ion mode, its excimer ion peak was 593.1505[M − H]^-^, and the elemental composition of the compound was C_27_H_30_O_15_. m/z 577.1563 was obtained by removing 1 molecule of hydroxyl from precursor ion, and *m*/*z* 415.1035 was obtained by removing 1 molecule. Finally, *m*/*z* 279.0874 was obtained by removing ring A from 1 to 3 bonds. After reviewing the literature and fragment information, it was inferred that the compound was rosacein b, and the fragmentation process was shown in Fig. 2A.

#### 3.3.2 Triterpenoid

Five triterpenoids were characterized. Taking compound 46 as an example, in the negative ion mode, its excimer ion peak was 821.3949[M − H]^-^, and the elemental composition of the compound was C_42_H_62_O_16_. m/z 777.4067 was obtained by removing 1 molecule of carboxyl group from the precursor ion, or *m*/*z* 645.3644 and *m*/*z* 469.3323 were obtained by continuous removal of 2 molecules of glucuronic acid. By referring to literature and fragment information, it was concluded that the compound was glycyrrhizic acid, and the fragmentation process was shown in Fig. 2B.

#### 3.3.3 Alkaloids

Five alkaloid compounds were characterized. Taking compound 5 as an example, the excimer ion peak was 268.1057[M+H]^+^ in the positive ion mode, and the elemental composition of the compound was C_10_H_13_N_5_O_4_. The removal of 1 molecule of ribose from precursor ion yields *m*/*z* 136.0618 or the removal of 1 molecule of amino acid yielded *m*/*z* 253.0931. After reviewing the literature and fragment information, it was concluded that the compound was adenosine, and the fragmentation process was shown in Fig. 2C.

#### 3.3.4 Glycoside

A total of 4 glycosides were characterized. Taking compound 19 as an example, in the negative ion mode, its excimer ion peak was 503.1618[M − H]^-^, and the elemental composition of the compound was C_18_H_32_O_16_. The precursor ion removed 2 molecules of glucose successively, and thus *m*/*z* 341.1089 and *m*/*z* 179.0661 were obtained. After reviewing the literature and fragment information, it was inferred that the compound was β-amylase, and the fragmentation process was shown in Fig. 2D.

### 3.4 The effect of DDO on splenic lymphocyte proliferation in vitro

**Fig. 3:**
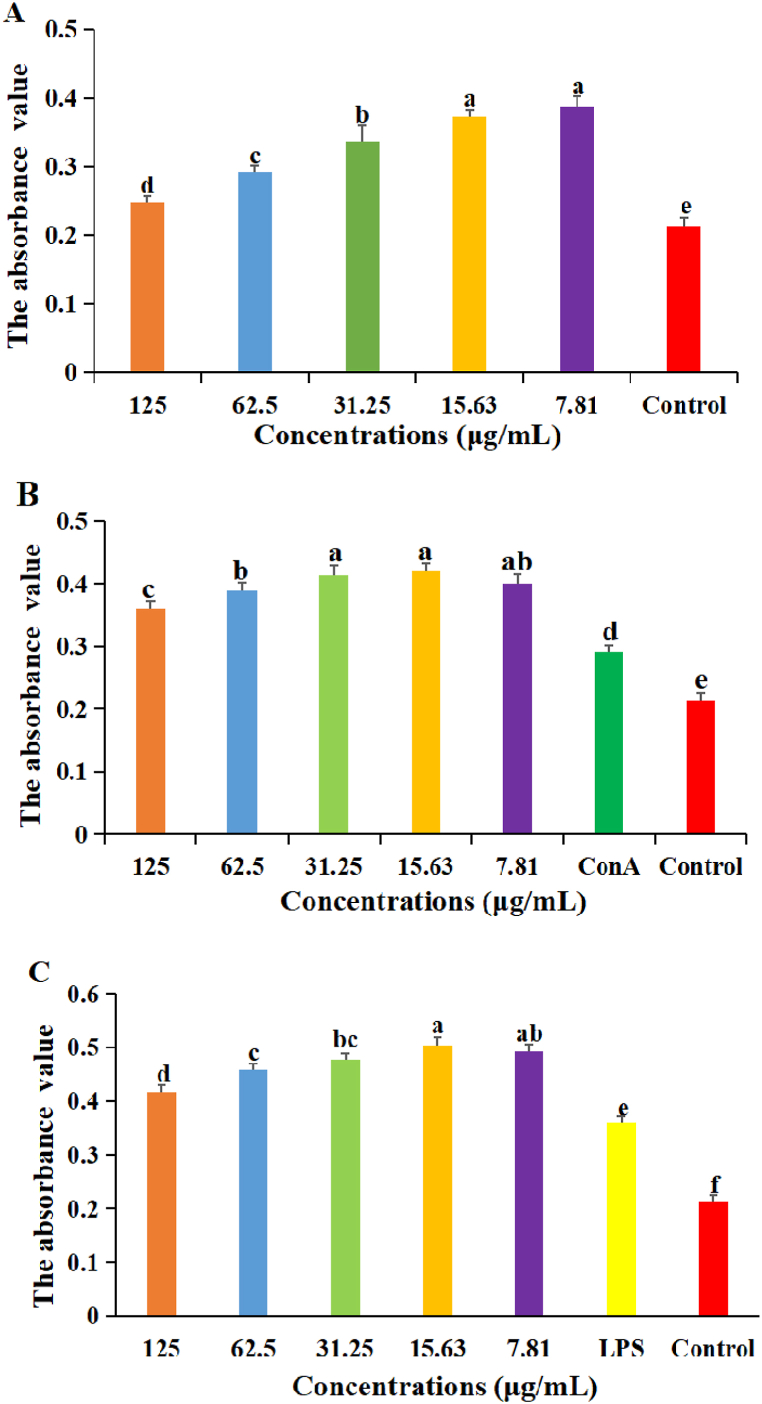
The effect of DDO on lymphocyte proliferation. (A) the lymphocyte proliferation in single stimulation of DDO. (B) the lymphocyte proliferation in synergistical stimulation of DDO with ConA. (C) the lymphocyte proliferation in synergistical stimulation of DDO with LPS. Data are expressed as the mean ± SD. ^a-f^ Bars in the figure without the same superscripts differ significantly (p < 0.05).

The results were shown in Fig. 3. When lymphocytes were stimulated with DDO, the A_570_ values of DDO at 125–7.81 μg/mL groups were significantly higher than that in control group (P < 0.05), the A_570_ values of DDO at 15.63 and 7.81 μg/mL groups were significantly higher than those in 125, 62.5 and 31.25 μg/mL groups (P < 0.05) (Fig. 3A).

When lymphocytes were synergistically stimulated with DDO and ConA, the A_570_ values of DDO at 125–7.81 μg/mL groups were significantly higher than those in ConA and control groups (P < 0.05), the A_570_ values of DDO at 31.25 and 15.63 μg/mL groups were significantly higher than those in 125 and 62.5 μg/mL groups (P < 0.05), there was no significant difference among 31.25, 15.63 and 7.81 μg/mL groups (Fig. 3B).

When lymphocytes were synergistically stimulated with DDO and LPS, the A_570_ values of DDO at 125–7.81 μg/mL groups were significantly higher than those in LPS and control groups (P < 0.05), the A_570_ values of DDO at 15.63 μg/mL group was the highest and significantly higher than those in 125, 62.5 and 31.25 μg/mL groups (P < 0.05) (Fig. 3C).

### 3.5 The effect of DDO on the phagocytosis of macrophages in vitro

**Fig. 4:**
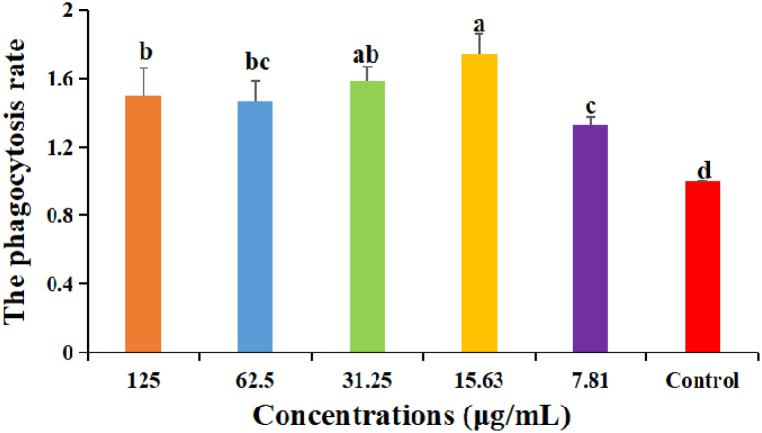
The effect of DDO on the phagocytosis of macrophages in vitro. Data are expressed as the mean ± SD. ^a-d^ Bars in the figure without the same superscripts differ significantly (p < 0.05).

The results were shown in Fig. 4. The phagocytosis rates of DDO at 125–7.81 μg/mL groups were significantly higher than that in control group (P < 0.05). The phagocytosis rate of DDO at 15.63 μg/mL group was the highest and significantly higher than those in 125, 62.5 and 7.81 μg/mL groups (P < 0.05).

### 3.6 The effect of DDO on the antibody-producing cells

**Fig. 5:**
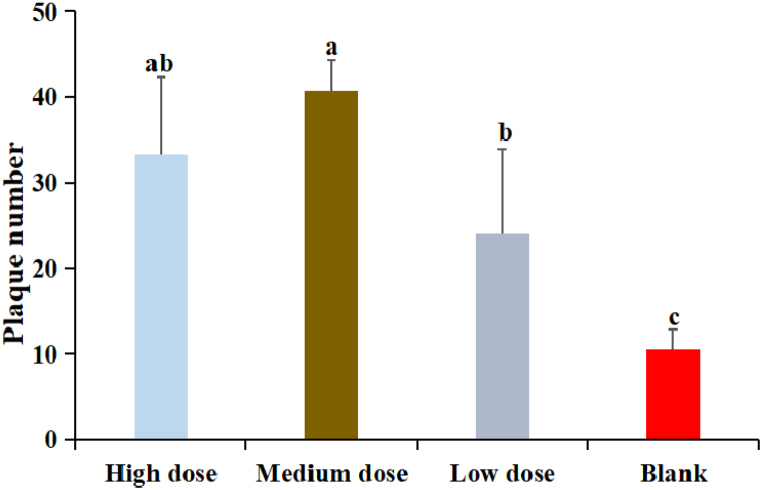
The effect of DDO on the antibody-producing cells. Data are expressed as the mean ± SD. ^a-c^ Bars in the figure without the same superscripts differ significantly (p < 0.05).

The results were shown in Fig. 5. The numbers of hemolytic plaque in DDO at three dose groups were significantly higher than that in blank group (P < 0.05). The number of plaque in DDO at medium dose group was the highest and significantly higher than that in low dose group (P < 0.05). There was no significant difference between high dose and medium dose groups (P > 0.05)

### 3.7 The effect of DDO on the NK cells activity

**Fig. 6:**
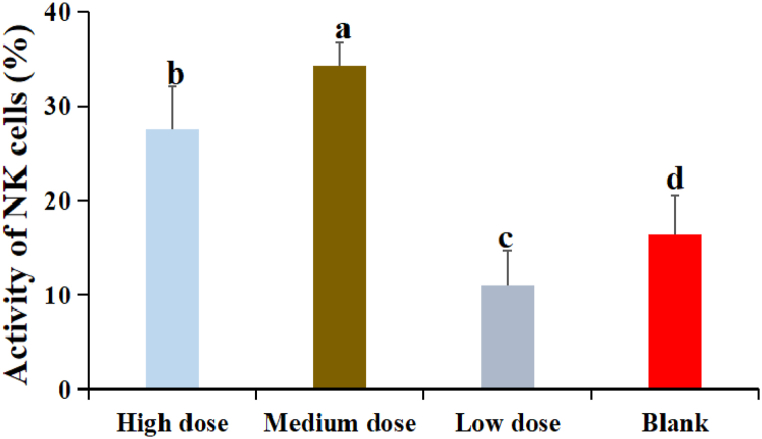
The effect of DDO on the NK cells activity. Data are expressed as the mean ± SD. ^a-f^ Bars in the figure without the same superscripts differ significantly (p < 0.05).

The results were shown in Fig. 6. The activity of NK cells in DDO at high and medium dose groups were significantly higher than that in low dose and blank groups (P < 0.05). The activity of NK cells in DDO at medium dose group was the highest and significantly higher than that other groups (P < 0.05).

### 3.8 The effect of DDO on the swelling rate of mice

**Fig. 7:**
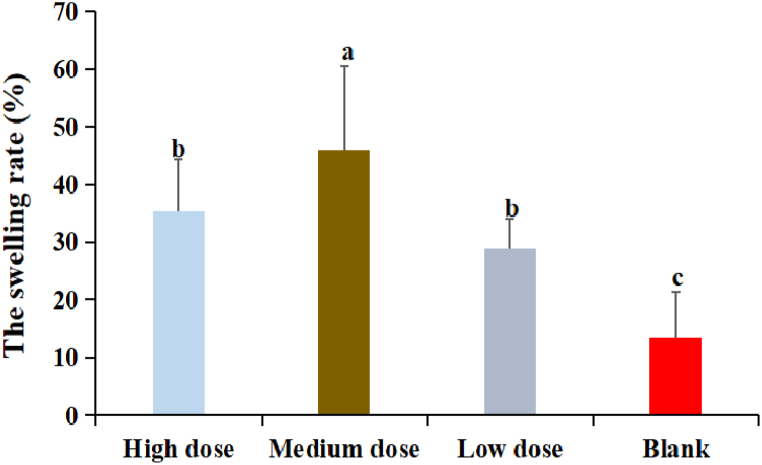
The effect of DDO on the swelling rate of mice. Data are expressed as the mean ± SD. ^a-f^ Bars in the figure without the same superscripts differ significantly (p < 0.05).

The results were shown in Fig. 7. The swelling rate in DDO at three dose groups were significantly higher than that in blank group (P < 0.05). The swelling rate in DDO at medium dose group was the highest and significantly higher than that in others group (P < 0.05). There was no significant difference between high dose and low dose groups (P > 0.05).

### 3.9 The effect of DDO on the phagocytosis index and immune organic index of mice

**Fig. 8:**
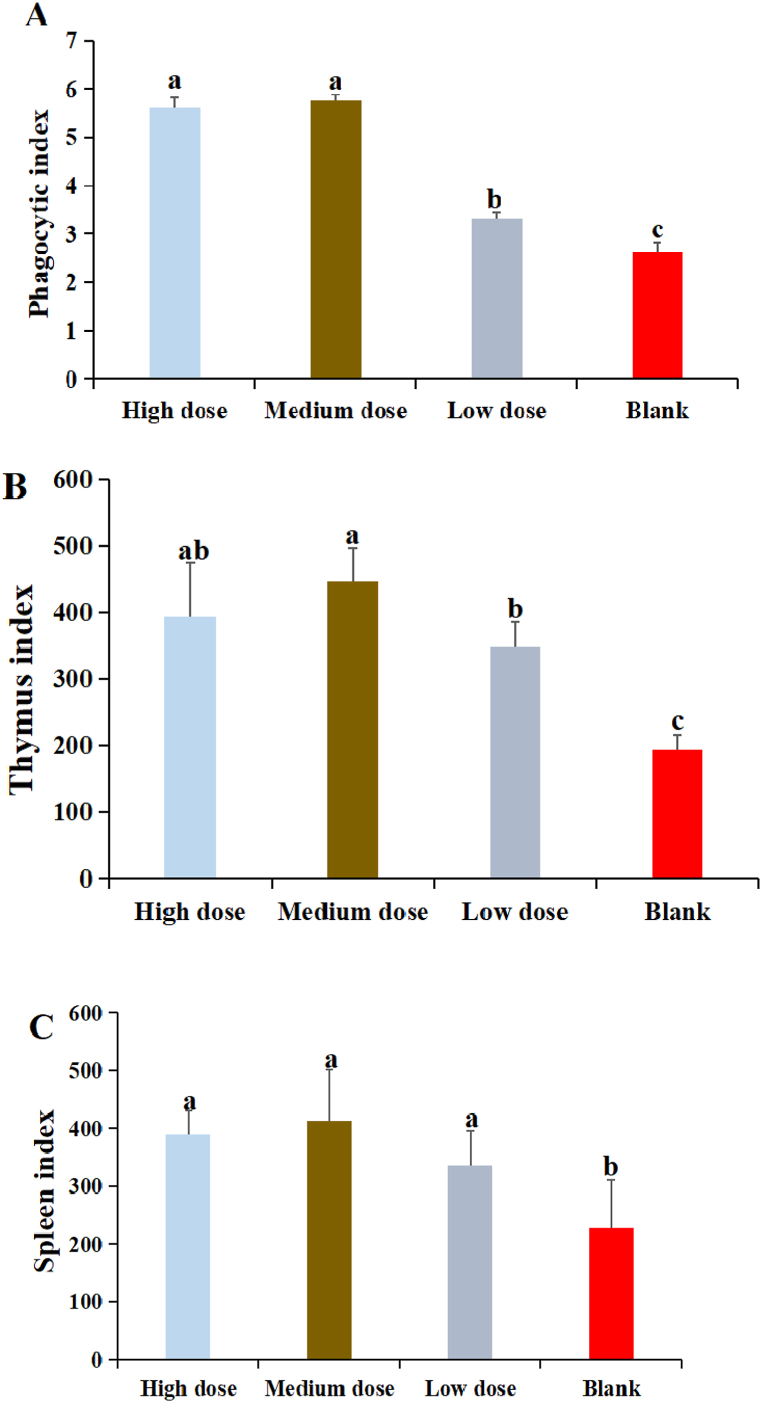
The effect of DDO on the phagocytosis index and immune organic index of mice. (A) The effect of DDO on the phagocytosis index. (B) The effect of DDO on the thymus index of mice. (C) The effect of DDO on the spleen index of mice. Data are expressed as the mean ± SD. ^a-c^ Bars in the figure without the same superscripts differ significantly (p < 0.05).

The results of DDO on the phagocytosis index were shown in Fig. 8A. The phagocytosis index in DDO at three dose groups were significantly higher than that in blank group (P < 0.05). The phagocytosis index in DDO at high and medium dose groups was significantly higher than that in low dose group (P < 0.05). There was no significant difference between high dose and medium dose groups (P > 0.05)

The results of DDO on the thymus index were shown in Fig. 8B. The thymus index in DDO at three dose groups were significantly higher than that in blank group (P < 0.05). The thymus index in DDO at medium dose group was the highest an significantly higher than that in low dose group (P < 0.05). There was no significant difference between high dose and medium dose groups (P > 0.05)

The results of DDO on the spleen index were shown in Fig. 8C. The spleen index in DDO at three dose groups were significantly higher than that in blank group (P < 0.05). There was no significant difference among three dose groups (P > 0.05)

### 3.10 The effect of DDO on splenic lymphocyte proliferation in vivo

**Fig. 9:**
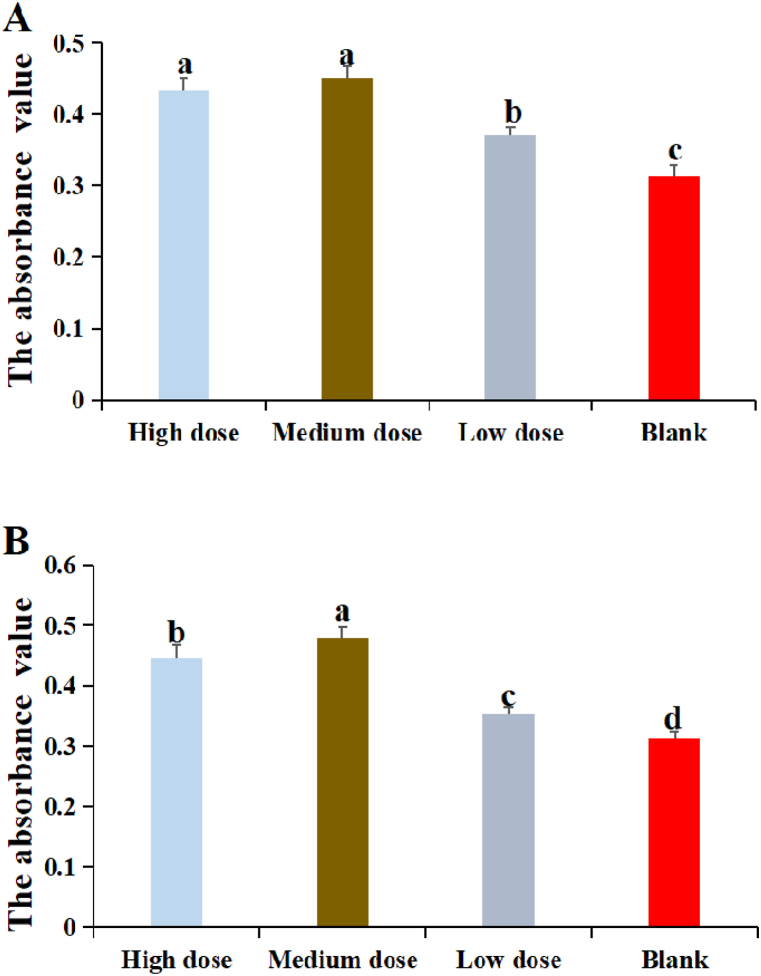
The effect of DDO on lymphocyte proliferation in vivo. (A) The lymphocyte proliferation in stimulation of ConA. (B) The lymphocyte proliferation in stimulation of LPS. Data are expressed as the mean ± SD. ^a-d^ Bars in the figure without the same superscripts differ significantly (p < 0.05).

**Fig. 10:**
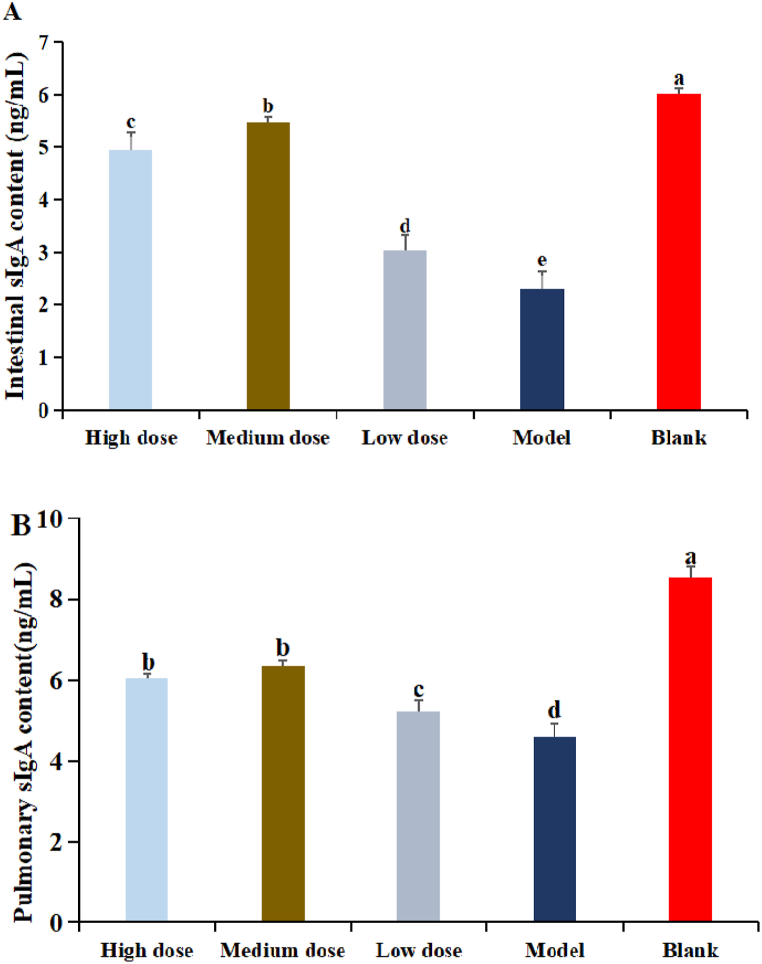
The effect of DDO on the level of sIgA in mice. (A) The content of sIgA in intestine of mice. (B) the content of sIgA in lung of mice. Data are expressed as the mean ± SD. ^a-e^ Bars in the figure without the same superscripts differ significantly (p < 0.05).

The results were shown in Fig. 10. When lymphocytes were stimulated with ConA, the A_570_ values of DDO at three dose groups were significantly higher than that in blank group (P < 0.05), the A_570_ values of DDO at medium dose group was the highest and significantly higher than that in low dose group (P < 0.05). There was no significant difference between high and medium dose groups (Fig. 9A)

When lymphocytes were stimulated with LPS, the A_570_ values of DDO at three dose groups were significantly higher than that in blank group (P < 0.05), the A_570_ values of DDO at medium dose group was the highest and significantly higher than those high and low dose groups (P < 0.05) (Fig. 9B)

### 3.11 The effect of DDO on the level of sIgA in mice

The results of DDO on the content of sIgA in intestine were shown in Fig. 10A

The content of sIgA in DDO at three dose groups were significantly higher than that in model group, but significantly lower than blank group (P < 0.05). The content of sIgA in DDO at medium dose groups was the highest and significantly higher than that in high and low dose groups (P < 0.05). The content of sIgA in DDO at high dose groups was significantly higher than that in low dose group (P < 0.05)

The results of DDO on the content of sIgA in lung were shown in Fig. 10B. The content of sIgA in DDO at three dose groups were significantly higher than that in model group (P < 0.05). The content of sIgA in DDO at high and medium dose groups was significantly higher than that in low dose group (P < 0.05). There was no significant difference between high dose and medium dose groups (P > 0.05)

## 4 Discussion

The compatibility of different traditional Chinese medicines in prescription contributes to enhancing the efficiency [21]. The mechanism of prescription might be related to the changes of the content and form of chemicals caused by the interaction among ingredients [22]. For example, Zhi-Zi-Chi decoction was comprised of Gardenia jasminoides Ellis and Semen sojae preparatum, which generated two new kinds of chemicals (jasminoside A and jasminoside I) and eight components disappeared (such as genipin, crocin III and so on) compared with single medicine. Moreover, the contents of six kinds of components were increased after the compatibility [23]. Therefore, the composition of DDO is not only the sum of three single medicines, so it was necessary to further investigate the compound changes after compatibility. At present, UPLC-Q-TOF/MS technology was widely used to analyze the medicine components because of its great efficiency and the potency for structural characterization [24]. Therefore, UPLC-Q-TOF/MS was applied to analyze the compounds in DDO. The results showed that 51 compounds were identified. It contained 26 flavonoids, 5 triterpenoids, 5 alkaloids, 4 glycosides, 3 coumarins, 2 organic acids, 2 esters and 4 other compounds. These compounds may be the active ingredients of DDO for improving the immunity. However, this study only carried out the qualitative analysis, and the quantitative measurement of the bioactive ingredients will be completed in the future

Immune system is the body's defense against foreign antigenic substances. In the immune response, non-specific immunity and specific immune response usually play the role together. Among them, specific immunity includes cellular immunity and humoral immunity. The level of immune function directly affects the strength of the body's resistance to disease and the prognosis of the disease [25]. Lymphocytes macrophages and NK cells are the important immune cells, and to investigate their changes is the important methods to directly evaluate the effects of drugs on immune regulation of the body

Lymphocytes are produced by lymphoid organs. When stimulated by foreign antigens such as ConA, LPS and so on, mature lymphocytes can show transformation phenomena such as volume increase and division. B and T lymphocyte are both important for immunologic response in organism. T lymphocyte possesses mediating cellular immunity as well as adjusting immunity. B lymphocyte mainly participates in the humoral immunity. Proliferation of lymphocyte is the most immediate index of reflecting organic immunity [26]. The experimental results showed that the absorbance value of DDO group was significant than control group, which indicated that DDO could significantly promote the proliferation of lymphocytes in vitro and in vivo, and the medium dose possessed the best efficacy in vivo. Many studies have proved that traditional Chinese medicine and its ingredients and compounds had the better effect on promoting lymphocyte proliferation [26,27]

Macrophages, known as an important cell in non-specific immunity, are found throughout bodily tissues and play an important role in the immune system. Upon activation by foreign substances, such as bacteria and viruses, macrophages become more efficient in eliminating pathogens, in comparison to their dormant state [28]. Macrophages are involved in phagocytosis, secretion of inflammatory mediators, and T cell activation [29]. In addition, activated macrophage secrete cytotoxic proteins, which play a role in the destruction of cancer, viruses, and bacteria within cells ([30]). Known as phagocytes, the phagocytosis ability of macrophage could be used to judge the non-specific immune function of the body. The experimental results proved that DDO could significantly improve the phagocytosis ability of macrophage in vitro and in vivo by natural red and carbon clearance test. Many studies have showed that traditional Chinese medicine and its ingredients and compounds had the better effect on improve the phagocytosis ability of macrophage [8,31]

NK cells are the special class of lymphocytes in the immune system that can be activated to mediate cytotoxic activity and produce the high levels of cytokines and chemokines involved in non-specific antiviral defense mechanisms [32]. In addition, NK cells have broad prospects in improving hematopoietic and organ transplantation, promoting anti-tumor immunotherapy, and controlling inflammation and autoimmune diseases [33,34]. The experimental results showed that three dosage groups of DDO could significantly improve the activity of NK cells, suggesting that DDO could improve the immune capacity of the body by enhancing the activity of these immune-related cells, and played an important role in disease prevention

The hemolytic plaque assay of spleen was used to detect the function of antibody forming cells in animals. Mice are immunized by intraperitoneal injection of sheep red blood cells, which could induce the mouse lymphocytes to produce specific antibodies, with the participation of lymphocytes and complement, the antibodies co-incubation with sheep red blood cells could produce hemolytic phenomenon and form hemolytic plaque [15,35]. It reflects the ability of cells to secrete antibodies, which belongs to the humoral immune response [36]. The results showed that DDO could increase the number of hemolytic plaques formed by the splenic lymphocytes of mice. Because the increase in the number of hemolytic plaques was proportional to the amount of antibodies formed by cells, it was proved that DDO could increase the number of antibodies in mice, and thus improve the humoral immunity function of mice

Delayed-type hypersensitivity reaction is the cellular immune response induced by antigen in the body. After specific antigen is re-exposed to sensitized T cells under the action of antigen presenting cells, T cells are activated, proliferated and differentiated into effector T cells, which further clear the antigen and cause the inflammation in local tissues [37]. After stimulation, visible and palpable swelling began to appear at 24 h. The cellular immune function of the organism could be evaluated by measuring the degree of swelling and comparing it with unstimulation. The experimental results showed that DDO could improve the delayed-type hypersensitivity reaction of mice, suggesting that DDO could improve the cellular immune response

Mucosal immune system is an important part of the body's immune network, is an important link of the body to resist infection, which constitutes the body's first line of defense against infection. Mucosal immune system mainly plays a role by producing secretory IgA (SIgA), which can neutralize pathogens, toxins and other harmful substances in mucosal epithelium and capture pathogens in the intermucosal layer [38]. sIgA antibody can not only inhibit the adhesion of pathogenic microorganisms, but also obstruct the interaction between pathogenic microorganisms and epithelial cells through the form of cross-linking polymers. It is the biological barrier to isolate the combination of pathogenic microorganisms and epithelial cells, and can prevent pathogens from invading the body [39,40]. In this study, sIgA antibody levels in the small intestinal mucosa and pulmonary mucosa of mice were measured. The results showed that DDO could stimulate the production of sIgA antibody in immunized mice. This suggested that DDO could stimulate mucosal immunity in mice

## 5 Conclusion

In this study, the chemical compounds of DDO were identified by UPLC-QTOF/MS method, and the immune activity was measured. The results showed that 51 compounds were identified. In addition, DDO could significantly improve the non-specific immunity and specific immune response. Although the effective material basis for DDO to improve immunity was clarified in this study, more researches are required for further explanation for the potential mechanism of different components in DDO.

## Funding

The project was supported by 10.13039/501100004735Hunan Provincial Natural Science Foundation of China (2021JJ40279) and Scientific Research Project of Hunan Provincial Health Commission (202113011086).

## Data availability statement

All data that supports the results were included in the article.

## Ethical approval

All experiments involving aquatic products in this study were conducted in accordance with the basic principles of experimentation and approved by the Animal Care and Use Committee of the Hunan Children's Hospital (HCHDWLL-2021-10).

## CRediT authorship contribution statement

**Zhihong Zhou:** Writing – original draft, Supervision, Project administration, Funding acquisition, Conceptualization. **Minzhuo Liu:** Writing – review & editing. **Xin Zhao:** Methodology, Formal analysis. **Haixia Li:** Methodology, Formal analysis. **Qin Hu:** Writing – review & editing. **Zhiping Jiang:** Validation, Data curation.

## Declaration of competing interest

The authors declare the following financial interests/personal relationships which may be considered as potential competing interestsZhihong Zhou reports financial support was provided by 10.13039/501100004735Hunan Provincial Natural Science Foundation of China. Zhihong Zhou reports financial support was provided by Scientific Research Project of Hunan Provincial Health Commission. If there are other authors, they declare that they have no known competing financial interests or personal relationships that could have appeared to influence the work reported in this paper.
